# Chromosome-scale genome assembly of *Apocynum pictum*, a drought-tolerant medicinal plant from the Tarim Basin

**DOI:** 10.1093/g3journal/jkae237

**Published:** 2024-11-05

**Authors:** Wenlong Xie, Baowei Bai, Yanqin Wang

**Affiliations:** College of Life Science and Technology, Tarim University, Xingfu Road, Alar 843300, Xinjiang, P.R. China; Xinjiang Production & Construction Corps Key Laboratory of Protection and Utilization of Biological Resources in the Tarim Basin, Tarim University, Alar 843300, Xinjiang, P.R. China; College of Life Science and Technology, Tarim University, Xingfu Road, Alar 843300, Xinjiang, P.R. China; Xinjiang Production & Construction Corps Key Laboratory of Protection and Utilization of Biological Resources in the Tarim Basin, Tarim University, Alar 843300, Xinjiang, P.R. China; College of Life Science and Technology, Tarim University, Xingfu Road, Alar 843300, Xinjiang, P.R. China; Xinjiang Production & Construction Corps Key Laboratory of Protection and Utilization of Biological Resources in the Tarim Basin, Tarim University, Alar 843300, Xinjiang, P.R. China

**Keywords:** de novo genome assembly, *Apocynum pictum*, transcriptome, drought tolerance

## Abstract

*Apocynum pictum* Schrenk is a semishrub of the Apocynaceae family with a wide distribution throughout the Tarim Basin that holds significant ecological, medicinal, and economic values. Here, we report the assembly of its chromosome-level reference genome using Nanopore long-read, Illumina HiSeq paired-end, and high-throughput chromosome conformation capture sequencing. The final assembly is 225.32 Mb in length with a scaffold N50 of 19.64 Mb. It contains 23,147 protein-coding genes across 11 chromosomes, 21,148 of which (91.36%) have protein functional annotations. Comparative genomics analysis revealed that *A. pictum* diverged from the closely related species *Apocynum venetum* approximately 2.2 million years ago and has not undergone additional polyploidizations after the core eudicot WGT-γ event. Karyotype evolution analysis was used to characterize interchromosomal rearrangements in representative Apocynaceae species and revealed that several *A. pictum* chromosomes were derived entirely from single chromosomes of the ancestral eudicot karyotype. Finally, we identified 50 members of the well-known stress-responsive WRKY transcription factor family and used transcriptomic data to document changes in their expression at 2 stages of drought stress, identifying a number of promising candidate genes. Overall, this study provides high-quality genomic resources for evolutionary and comparative genomics of the Apocynaceae, as well as initial molecular insights into the drought adaptation of this valuable desert plant.

## Introduction

The Tarim Basin, situated in southern Xinjiang, is the largest inland basin in China. It is bordered by the Kunlun Mountains in the south, connected to the Tianshan Mountains in the north, and stretches from the Altun Mountains in the east to the Pamir Plateau in the west. In the central part of the basin lies the Taklamakan Desert. The Tarim Basin falls within the temperate zone and experiences a continental arid and semiarid climate. It frequently encounters dust storms and sandstorms and is characterized by long periods of sunshine and significant temperature fluctuations between day and night. Previous studies have concluded that a hyperarid climate has persisted within the basin for at least 5.3 million years, and there is evidence of increasing aridity (Sun et al. 2008). The basin's unique geographic location and challenging climate conditions contribute to a mere 10% vegetation coverage along its periphery, primarily composed of shrubs and semishrubs (Sun et al. 2008; Jiang et al. 2022). These vegetation types are excellent materials for studying plant adaptations to dry environments.

*Apocynum pictum* Schrenk (2*n* = 22, synonym of *Apocynum hendersonii* Hook. f.), commonly referred to as Bai ma, is a semishrub from the Apocynaceae family that grows predominantly in the northwestern inland region in areas surrounding saline-alkali wastelands, desert edges, and river alluvial plains. It exhibits a wide distribution throughout the Tarim Basin (Yang et al. 2021). *A. pictum* shows strong ecological adaptability to cold, drought, wind erosion, and saline-alkaline conditions, and it is often used as a windbreak and sand-fixing plant in Xinjiang, China. *A. pictum* also serves as a valuable source of honey and has economic significance as an excellent fiber plant because of the high quality of phloem fibers in its stems (Rouzi et al. 2017). In addition, *A. pictum* has been used as a pharmacological plant because of its significant content of flavonoids, primarily quercetin (Ma et al. 2003). Previous studies have focused on the closely related species *Apocynum venetum*, also called Luobuma, which has demonstrated a wide range of medicinal properties (Xie et al. 2012). Although recent developments in sequencing technology have enabled assembly of the chloroplast and nuclear genomes of *A. pictum* (Zheng et al. 2022; Gao et al. 2023), there is still a need for a high-quality, chromosome-scale reference genome.

In this study, we produced a chromosome-scale reference genome and annotations for *A. pictum* by integrating Oxford Nanopore Technologies (ONT) long-read, Illumina HiSeq paired-end, and high-throughput chromosome conformation capture (Hi-C) sequencing. We examined the general characteristics of *A. pictum* genome evolution, identified 50 members of the WRKY transcription factor (TF) family, and performed transcriptome sequencing at 2 stages of the drought stress response. The de novo assembly and annotation of this high-quality *A. pictum* genome provide a basis for future evolutionary research on the Apocynaceae family and support continued study and utilization of this versatile and economically valuable plant.

## Methods and materials

### Sample collection and sequencing

The individual *A. pictum* plant used for sequencing was growing in the northern region of the Tarim Basin (81.31E, 40.54N; Xinjiang, China); fresh shoots were collected and transported on dry ice to BIOYIGENE (Wuhan, China) for genome sequencing. Total genomic DNA was extracted using the QIAGEN Genomic DNA Extraction Kit. After repairing DNA damage, the purified DNA fragments were subjected to end repair and A-tailing reactions at both ends of the DNA fragment. Following purification, the DNA fragments were ligated with adapters obtained from the Ligation Sequencing Kit (SQK-LSK109). Long reads were sequenced on the PromethION platform. Low-quality reads were removed after basecalling (mean_qscore_template < 7), and short reads were removed using Filtlong v0.2.1 (−min_length 1,000). For short reads sequenced on the Illumina HiSeq 4000 platform, adapters and low-quality reads were removed using Trim Galore v0.6.7 (-q 25 -phred33 -length 100 -stringency 1 -paired). For Hi-C sequencing, tender shoots were fixed in a 1% formaldehyde solution to cross-link chromatin and then digested with the MboI restriction enzyme. A DNA library was constructed and sequenced on the Illumina HiSeq 4000 platform, and fastq sequencing files were trimmed with fastp (Chen et al. 2018) v0.12.6 using default parameters. We ultimately obtained 17 Gb of Illumina short-read sequencing data, 48 Gb of Hi-C sequencing data, and 21 Gb of ONT long-read sequencing data for use in assembly of the *A. pictum* genome scaffolds (Supplementary Table 1).

Transcriptome sequencing of fresh leaf and stem tissue from the same plant was performed for the purpose of gene structural annotation. RNA extraction, library construction, and sequencing were performed by BIOYIGENE (Wuhan, China) on the MGI2000 platform. For samples from the drought experiment (see below), total RNA was extracted from leaf tissue using the QIAGEN RNAprep pure Plant Kit. Paired-end library preparation and quality control were performed, and libraries were sequenced on the MGISEQ-2000RS platform at the High-Throughput Sequencing Platform of the National Key Laboratory of Crop Genetic Improvement at Huazhong Agricultural University (Wuhan, China).

### Genome survey and de novo assembly

We used Illumina data to estimate the genome size, heterozygosity ratio, and repetitive sequence content of *A. pictum* using *k*-mer distribution analysis (*k* = 17). We first used jellyfish v2.3.0 (-m 17 -C -s 300 M) to count and compute the frequency of 17-mers and then visualized the 17-mer count histogram using GenomeScope2 (Ranallo-Benavidez et al. 2020) v2.0. We then generated a contig-level assembly of ONT reads using NextDenovo (Hu et al. 2024) v2.5.0 (genome size = 202 m, read_cutoff = 1k, correction_options = -p 15 -dbuf) and performed 2 rounds of polishing with Illumina short reads using NextPolish (Hu et al. 2020) v1.4.1 with default parameters. To improve the genome contiguity and obtain a chromosome-level contig assembly, we used a combination of Juicer v2.0 (-s MboI –assembly) and 3D-DNA (Dudchenko et al. 2017) (2 rounds of polishing with default parameters) strategies with the Hi-C sequencing data. We used Juicebox Assembly Tools (Durand et al. 2016) to manually refine the chromosome boundaries and then executed the run-asm-pipeline-post-review.sh script in the 3D-DNA pipeline to obtain the chromosome-level assembly. We examined the pseudochromosome interactions in a Hi-C heatmap using plotHicGenome software and made manual adjustments to chromosome order and orientation.

We used multiple methods to evaluate the completeness and quality of the assembled genome, including Benchmarking Universal Single-Copy Orthologs (BUSCO) (Simão et al. 2015) v5.3.2 with the eudicots_odb10 database (Supplementary Table 2) and calculation of the long terminal repeat (LTR) assembly index (LAI) value (Ou et al. 2018). LTR_Finder (Xu and Wang 2007) v1.0.7 (-D 15000 -d 1000 -L 7000 -l 100 -p 20 -C -M 0.85) and LTRharvest (Ellinghaus, Kurtz, and Willhoeft 2008) (-minlenltr 100 -maxlenltr 7000 -mintsd 4 -maxtsd 6 -motif TGCA -motifmis 1 -similar 85 -vic 10) were used to predict LTR retrotransposons (LTR-RTs) and construct an LTR sequence library. The LAI value was then calculated using the perl program LTR_retriever (Ou and Jiang 2018) with default parameters. We also used the Quality Assessment Tool (Gurevich et al. 2013) v5.2.0 to align Illumina short reads to the draft genome and obtain genome statistics.

To evaluate genome base accuracy, we called SNPs and indels using the single-sample workflow of the Genome Analysis Toolkit (McKenna et al. 2010) (GATK) v4.3.0 pipeline (Supplementary Table 3). SAMtools v1.6 and bwa-mem2 v2.2.1 were used to build an index of the draft genome, and the GATK CreateSequenceDictionary script was used to create the sequence dictionary. The fastq file of Illumina short reads was converted to uBAM format with the GATK FastqToSam algorithm and then aligned to the draft genome to obtain a clean BAM file after marking Illumina adapters and duplicate sequences. A variant call format file was generated after processing using the GATK HaplotypeCaller algorithm. SNPs and indels were identified using standard hard-filtering parameters (O'Connor and van der Auwera 2020). The following parameters were used for SNPs: QD < 2.0 || MQ < 40.0 || FS > 60.0 || SOR > 3.0 || MQRankSum < −12.5 || ReadPosRankSum < −8.0. For Indels, the parameters used were as follows: QD < 2.0 || FS > 200.0 || SOR > 10.0 || MQRankSum < −12.5 || ReadPosRankSum < −8.0.

### Repetitive element and structural annotations

We identified tandem repeats using Tandem repeats finder (Benson 1999) with the parameters “2 7 7 80 10 50 500 -f -d -m -r -h” (Supplementary Table 4). Transposable element (TE) annotations were based on a custom repeat library that included a de novo repeat library generated with RepeatModeler (Flynn et al. 2020) v2.0.3, the lamiids and ancestral consensus repeats from the Repbase (Jurka et al. 2005) v20181026 database, the Dfam (Storer et al. 2021) database, and the LTR sequence library used to calculate the LAI score. We used RepeatMasker v4.1.2 with the custom repeat library to identify repetitive elements in the genome, and we obtained the soft-masked genome with the following parameters: -nolow -no_is -engine ncbi -gff -norna -xsmall -poly (Supplementary Table 5).

To annotate noncoding RNAs (ncRNAs), we used the cmscan program (–cut_ga –rfam –nohmmonly –fmt 2) in Infernal (Nawrocki and Eddy 2013) v1.1.4 to search for ncRNA sequences against the Rfam (Kalvari et al. 2020) v14.10 database with the cmpress program. We primarily retained hits that did not overlap with any other hits or, in the case of overlapping hits, those that had a lower *E*-value compared with the others (Supplementary Table 6).

We performed structural annotation using the BRAKER (Gabriel et al. 2024) v3.0.2 (ETPmode) automated annotation pipeline (Hoff et al. 2019). We used a custom protein database consisting of 2 closely related species, *Coffea arabica* (GCF_003713225.1) and *Coffea canephora* (GCA_900059795.1), for homologous prediction, and we incorporated orthologous proteins from the Viridiplantae in the OrthoDB (Kuznetsov et al. 2023) v10 database. The custom database and 26 Gb of paired-end RNA-sequencing data were used to train GeneMark-ETP (Bruna, Lomsadze, and Borodovsky 2020) to provide supporting evidence. The soft-masked genome was then used to train AUGUSTUS (Stanke et al. 2006) for de novo annotation predictions using the GeneMark-ETP results (Supplementary Table 7). The final functional gene set was filtered using TEsorter (Zhang et al. 2022) v1.4.6 (-eval 1e-6), taking into consideration that these genes may have been inactivated by transposon insertion. The quality of annotations was evaluated using BUSCO (Simão et al. 2015) in protein mode (Supplementary Table 2), and the predicted proteins were functionally annotated using the EggNOG v5.0 database (Huerta-Cepas et al. 2019).

### Phylogenetic analysis

The longest transcript from 10 species was used to identify orthologous gene families using OrthoFinder (Emms and Kelly 2019) v2.5.5 (-M msa), with multiple sequence alignment specified through Diamond (Buchfink, Reuter, and Drost 2021) v2.1.8.162 as the method for maximum likelihood species tree inference (Emms and Kelly 2018). Divergence times for single-copy orthologs were estimated using the MCMCtree program from the PAML v4.10.6 package (Yang 2007; Reis and Yang 2011). The species tree was calibrated using 2 fossil constraints from the TimeTree (Kumar et al. 2017) website: the estimated divergence of *Oryza sativa* and *Arabidopsis thaliana* at 142.1–163.5 million years ago (MYA) and that of *A. thaliana* and *Vitis vinifera* at 109.8–124.4 MYA. Expansion and contraction of gene families were analyzed using CAFE5 (Mendes et al. 2020) (-p -k 5) to estimate evolutionary rates under a birth–death model using maximum likelihood estimation. Expanded gene families in *A. pictum* were subjected to Gene Ontology (GO) and Kyoto Encyclopedia of Genes and Genomes (KEGG) enrichment analyses using the ClusterProfiler (Wu et al. 2021) v4.2.2 R package with the annotation file to predict their putative functions and pathways. Results of phylogenetic analysis were visualized using ChiPlot (Xie et al. 2023).

### Whole-genome duplication analysis

Paralogous and orthologous genes within and between genomes were identified using an all-against-all homology search performed with BLASTP (Camacho et al. 2009) using an *E*-value cutoff of 1e−5. Syntenic blocks within and between genomes were detected using MCscanX (Wang et al. 2012) with the criterion that at least 5 gene pairs were retained. MAFFT (Katoh and Standley 2013) v7.310 multiple sequence alignment of paralogous and orthologous genes was performed with WGDI (Sun et al. 2022) software, and the coding sequences of these genes were used to estimate nonsynonymous (Ka) and synonymous (Ks) substitution rates. The NeiGojobori method implemented in the YN00 program provided by PAML was used for this purpose (Yang 2007). Whole-genome duplication (WGD) events were detected by Gaussian fitting of the Ks distribution using WGDI software with the parameters: -bi -kp -pf -kf. Syntenic blocks between chromosomes were visualized with the bar plot function of WGDI using the parameters -km and -k.

### Identification of *WRKY* genes

The sequences of WRKY proteins from *A. thaliana* were obtained from the TAIR (Berardini et al. 2015) database. A hidden Markov model search against the *A. pictum* genome was performed using the PFAM (Mistry et al. 2021) database and the CDD (Wang et al. 2023) database to identify candidate WRKY genes. Only sequences containing the WRKY domain (PF03106) were retained as candidates. The chromosomal distribution of *ApWRKY* genes was displayed using TBtools (Chen et al. 2020) software. From a multiple sequence alignment obtained with ClustalW using clearly classified *AtWRKYs*, we constructed a neighbor-joining phylogenetic tree using MEGA X (Kumar et al. 2018) with 1,000 bootstrap replicates. The ApWRKY protein sequences were submitted to MEME Suite v5.5.5 to identify up to 10 conserved motifs, and these results were integrated with the CDD search results and gene structure file using TBtools software. Protein properties of the *ApWRKYs*, including length, molecular weight, pI, instability index, aliphatic index, and GRAVY score, were predicted using ExPASY (Supplementary Table 10). The 2,000-bp sequence upstream of the translation initiation sites of the *ApWRKY* genes was extracted using TBtools and then submitted to the PlantCARE (Lescot et al. 2002) database to predict potential *cis*-acting regulatory elements. The results were visualized using R scripts.

### Transcriptome analysis

The healthy seedlings of *A. pictum* were treated with 50 mL of 30% polyethylene glycol (PEG) 6,000 to simulate drought stress conditions throughout their 4-week growth period. Drought stress was divided into 2 stages: early (4 h) and late (12 h), and each treatment replicated for 3 times. Leaf tissues collected from *A. pictum* under 0, 4, and 12 h drought stress were used to prepare Illumina sequencing libraries as described above. Adapters and low-quality reads were removed from the resulting sequencing data using fastp (Chen et al. 2018) v0.12.6 with default parameters. The clean reads were mapped to the *A. pictum* genome, and raw count values were calculated using STAR (Dobin et al. 2013) v2.5.2 (–quantMode GeneCounts). Differentially expressed genes (DEGs) were defined as those exhibiting at least 1-fold change in expression compared to the 0-h time point and were identified using the DESeq2 R package, with an FDR-adjusted *P* < 0.05. To investigate the expression patterns of DEGs at 2 time points, we transformed the raw count data into transcripts per kilobase of exon model per million mapped reads (TPM). Cluster analysis of gene expression patterns was performed with the Mfuzz (Kumar and Futschik 2007) v2.54.0 R package; the results were used to construct an expression heatmap and perform KEGG/GO enrichment analysis using the ClusterGvis R package.

## Results

### Genome survey, sequencing, assembly, and assessment

**Fig. 1.**
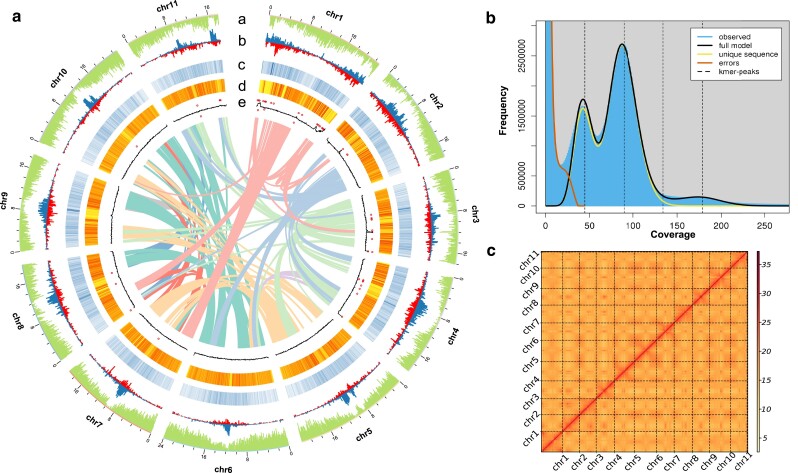
a) *Landscape* of the *A. pictum* genome assembly and annotation. Tracks from outside to inside correspond to a, gene density; b, Copia density and Gypsy density, respectively; c, repeat density; d, SNP density; e, GC density and N ratio. Genome features are depicted in 10-kb windows across the chromosomes. b) 17-mer depth distribution. The genome was estimated to be 202 Mb in size, with a heterozygosity rate of 1.13% and a repeat percentage of 37%. c) Hi-C assisted assembly of *A. pictum* pseudochromosomes. The heatmap reveals the presence of an antidiagonal pattern of intrachromosomal interactions, which were scaffolded and assembled independently.

We estimated the genome size of *A. pictum* by analyzing the distribution of 17-mers using 16.98 Gb of Illumina HiSeq data (Fig. 1b) and and then used a combination of ONT, Illumina HiSeq, and Hi-C data to assemble the *A. pictum* genome (Supplementary Table 1). 21.49 Gb of ONT long reads, with a coverage of 95×, were utilized for self-correction and initial contig assembly. Subsequently, the resulting assembly underwent two rounds of polishing with Illumina short reads. This produced a preliminary assembly of 59 contigs with a contig N50 of 9.63 Mb (Table 1). We used 48.26 Gb of Hi-C data to obtain 26 scaffolds with a scaffold N50 of 19.64 Mb, which enabled us to anchor the contigs to 11 pseudochromosomes (Fig. 1a and c). Of the contigs, 99.55% were successfully anchored to pseudochromosomes, producing a final assembled genome of 225 Mb.

**Table 1.** Statistics for A. pictum genome assembly and annotation.

Feature	Value
Genome size (bp)	225,320,346
GC content (%)	32.44
Number of contigs	59
Contig N50 (bp)	9,635,369
Contig N90 (bp)	3,354,232
Number of scaffolds	26
Scaffold N50 (bp)	19,644,087
Scaffold N90 (bp)	18,080,797
Sequence anchored to pseudochromosomes (bp)	224,312,087
LAI	15.37
Repetitive sequences (%)	35.95
Gene number	23,147
Average gene length (bp)	3,574

The completeness of the assembled genome was assessed using BUSCO (Simão et al. 2015). Of the 2,326 BUSCO orthologs in the eudicots database, 2,250 (96.8%) were completely captured in the genome (Supplementary Table 2). Of the Illumina short reads, 93.41% could be mapped to the genome. The assembly accuracy rate based on the percentage of homozygous single-nucleotide variants was 99.99% (Supplementary Table 3). The LAI of the *A. pictum* genome was 15.37.

### Repetitive element and gene annotations

A total of 35.95% (80.99 Mb) of the assembly was identified as repetitive elements using both de novo and homology-based methods (Table 1). This included tandem repeats, which represented 2.64% (5.94 Mb) of the total genome (Supplementary Table 4). Another category comprised elements that were dispersed across the genome, primarily TEs, which represented 22.35% (50.36 Mb) of the genome. Among the LTR-RTs of the class I TEs, *Gypsy* (9.96%) and *Copia* (8.69%) were the most prevalent superfamilies (Supplementary Table 5). We also identified 1,827 noncoding RNAs in the *A. pictum* genome, including 85 miRNAs, 549 snRNAs, 416 tRNAs, and 688 rRNAs (Supplementary Table 6).

We used transcriptome-based, homology-based, and de novo strategies to predict protein-coding genes in the assembled genome. A total of 23,147 protein-coding genes were identified, with an average length of 3,574 bp and an average CDS length of 1,303 bp (Supplementary Table 7). Most (21,148; 91.36%) of the predicted protein-coding genes were functionally annotated using the EggNOG (Huerta-Cepas et al. 2019) database, and 81.38% contained conserved protein domains as determined using the PFAM (Mistry et al. 2021) database (Supplementary Table 8). We assessed the quality of the protein-coding gene annotations using BUSCO and obtained 2,232 (96.0%) of the complete BUSCO orthologs (Supplementary Table 2).

### Phylogenetic analysis

**Fig. 2.**
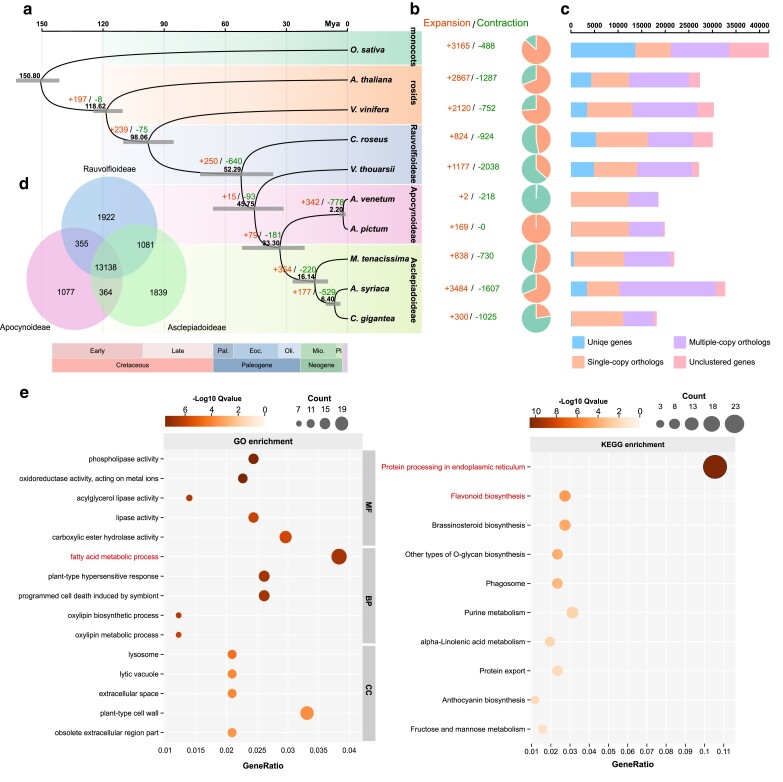
a) Maximum likelihood phylogenetic tree constructed from 1,376 single-copy orthologs and divergence time estimates for *A. pictum* and other species, rooted with *O. sativa* as the outgroup. b) Expansions and contractions of gene families in each species. c) Numbers of unique, single-copy, and multi-copy genes in *A. pictum* and other species. d) Venn diagram of unique and shared orthologous genes among Rauvolfioideae, Apocynoideae, and Asclepiadoideae. e) GO and KEGG enrichment analyses of expanded gene families in *A. pictum*, related to the common ancestor of the *A. pictum* and *A. venetum*. The top 5 significantly enriched biological process, cellular component, and molecular function GO terms are shown at left and the top 10 significantly enriched KEGG pathways at right. Dot color represents the *Q*-value, and dot size represents the number of genes mapped to the indicated terms/pathways.

We performed maximum likelihood phylogenetic analyses using protein sequences from 10 species: 2 from Apocynoideae (*A. pictum* and *A. venetum*), 2 from Rauvolfioideae [*Catharanthus roseus* (Xu et al. 2023) and *Voacanga thouarsii* (Cuello et al. 2022)], 3 from Asclepiadoideae [*Marsdenia tenacissima* (Zhou et al. 2023), *Asclepias syriaca* (Weitemier et al. 2019b), and *Calotropis gigantea* (Hoopes et al. 2018)], 2 from the Rosids (*A. thaliana* and *V. vinifera*), and 1 from the monocots (*O. sativa*) as an outgroup species (Supplementary Table 9). Here, we examined the evolutionary patterns of representative Apocynaceae species based on a phylogenetic tree derived from 1,376 single-copy orthologs (Fig. 2a).

Asclepiadoideae appeared to have diverged from a hypothetical common ancestor approximately 33.3 MYA. This divergence time aligns with the fossil evidence, in which the earliest record of Asclepiadoideae dates back to the Eocene epoch (33.9–56 MYA) (Del Rio et al. 2020). Asclepiadoideae and Apocynoideae are part of the APSA clade (Apocynoideae, Periplocoideae, Secamonoideae, and Asclepiadoideae); we estimated the age of the crown node for the APSA at 45.75 MYA, consistent with estimates from previous reports (45–65 MYA) (Ribeiro et al. 2014; Fishbein et al. 2018). On the basis of protein sequence homology, 244,695 (91.3%) genes were clustered into 24,459 gene families; 13,138 families were shared by subfamilies of the Apocynaceae, and 1,077 families were specific to the Asclepiadoideae (Fig. 2c and d).

*A. pictum* and *A. venetum* display close genetic relationships. In comparison to their ancestor, *A. pictum* showed an expansion of 169 gene families, and *A. venetum* exhibited a contraction of 218 gene families and expansion of 2 gene families (Fig. 2b). We examined the functions of the expanded gene families in *A. pictum* by GO and KEGG enrichment analyses (Fig. 2e).

### WGD analyses and karyotype evolution

**Fig. 3.**
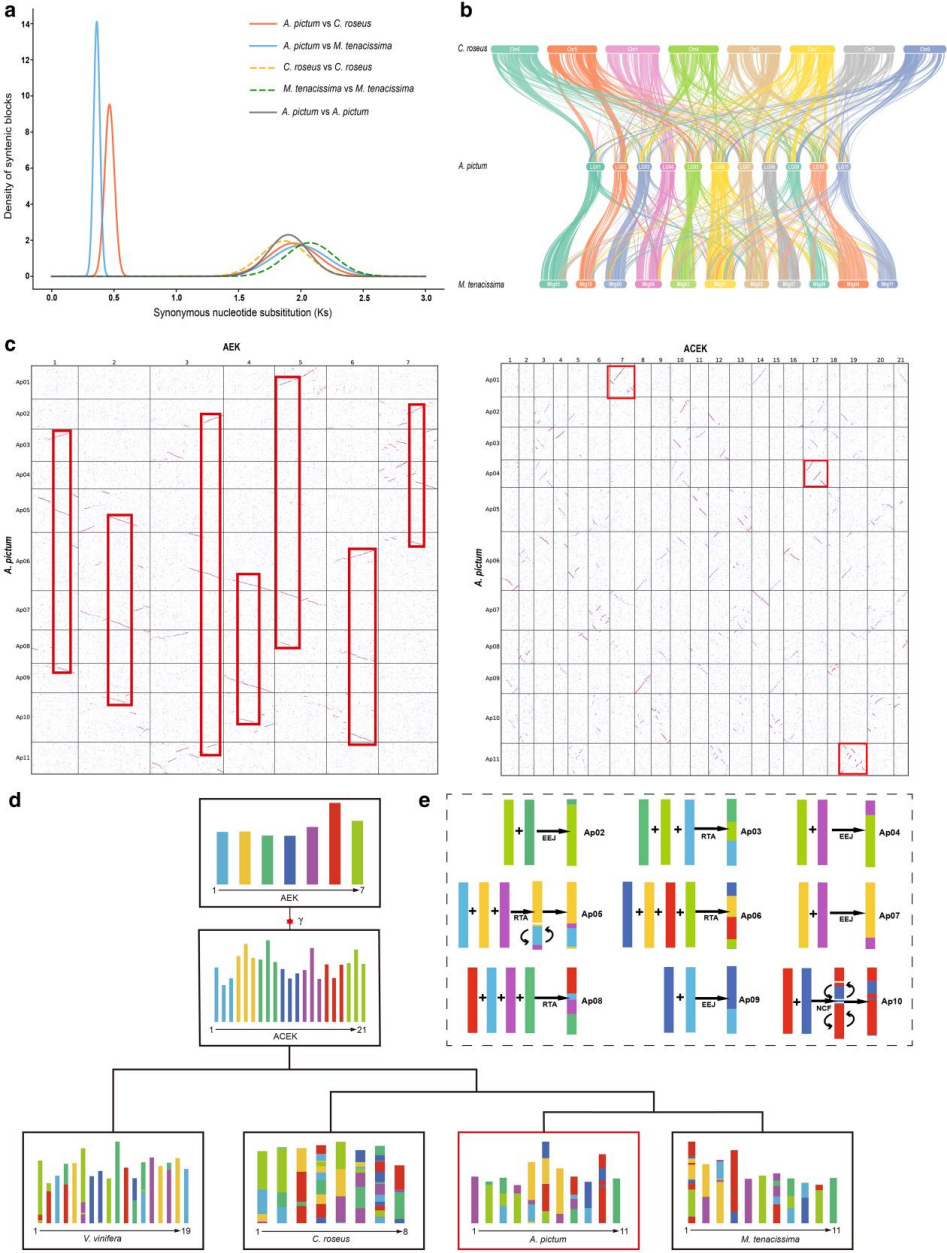
a) Synonymous nucleotide substitution (Ks) distributions for different pairs of *A. pictum*, *C. roseus*, and *M. tenacissima*. *A. pictum* and *M. tenacissima* divergence at Ks = 0.36; *A. pictum* and *C. roseus* divergence at Ks = 0.46. Three species shared WGT-γ event at Ks = 1.95. b) Gene synteny between *A. pictum*, *C. roseus*, and *M. tenacissima*. c) Syntenic block dotplots between *A. pictum* and the AEK (depth ratio 3:1) and between *A. pictum* and the ACEK (depth ratio 1:1). Red lines represent high-confidence syntenic blocks. Chromosomes 1, 4, and 11 of *A. pictum* are almost entirely derived from chromosomes 7, 17, and 19 of the ancestral ACEK. d) Karyotype projections of *V. vinifera*, *C. roseus*, *A. pictum*, and *M. tenacissima.* The AEK gave rise to the ACEK through the WGT-γ event. e) Proposed history of karyotype evolution in *A. pictum*. RTA, reciprocally translocated chromosome arms; EEJ, end-to-end joining; NCF, nested chromosome fusion.

We used Ks substitution rates to investigate the polyploidization histories of *A. pictum* and 2 other Apocynaceae species with high-quality chromosome-scale genome assemblies, *M. tenacissima* (Zhou et al. 2023) from the Asclepiadoideae and *C. roseus* (Xu et al. 2023) from the Rauvolfioideae (Fig. 3a). We estimated the Ks distribution of orthologous gene pairs in intergenomic syntenic blocks and observed distinct narrow peaks at 0.36 and 0.46, suggesting that *M. tenacissima* diverged from the common ancestor after the divergence of *A. pictum* and *C. roseus*. In genomic synteny plots of the 3 species (Fig. 3b), the individual chromosomes of *A. pictum* exhibited the most significant matches with corresponding chromosomes of *M. tenacissima*, suggesting a closer genetic relationship of *A. pictum* with *M. tenacissima* than with *C. roseus.* When we estimated the Ks distribution of paralogous gene pairs in intragenomic syntenic blocks, we detected a single broad peak at approximately 1.95, indicating that a single polyploidization event was shared by all 3 species.

To characterize the WGD event in the evolutionary history of *A. pictum*, we performed karyotype assessments using the ancestral eudicot karyotype (AEK) and the ancestral core eudicot karyotype (ACEK) as references (Wang et al. 2022). We mapped the *A. pictum* genome onto the AEK and ACEK genomes and generated syntenic block dotplots (Fig. 3c). The syntenic depths were determined to be 3:1 and 1:1, respectively. The chromosomes 1, 4, and 11 of *A. pictum* originated from ancestral chromosomes 7, 17, and 19 of the ACEK (Fig. 3c).

We next performed a karyotype evolution analysis to examine details of chromosome reorganization in representative Apocynaceae (Fig. 3d). We identified 335, 368, and 354 homologous blocks between *A. pictum* and the AEK, *C. roseus* and the AEK, and *M. tenacissima* and the AEK, respectively. In Fig. 3d, chromosomal regions of extant species that are homologous to portions of the AEK chromosomes are indicated with corresponding colors. *C. roseus* has undergone more chromosome fusions than *A. pictum* and *M. tenacissima*. Every chromosome in *C. roseus* originated from multiple AEK ancestral chromosomes, whereas chromosomes 1 and 11 in *A. pictum* and chromosomes 5, 6, and 11 in *M. tenacissima* originated from single AEK ancestral chromosomes. Fusion is the predominant form of interchromosomal rearrangement, encompassing reciprocally translocated chromosome arms (RTAs), end-to-end joining (EEJ), and nested chromosome fusion (NCF). The dotplot and karyotype projection of *A. pictum* and the AEK reveal that chromosomes 2, 4, 7, and 9 in *A. pictum* were formed through EEJ from AEK protochromosomes; chromosomes 3, 5, 6, and 8 were formed through RTA; and chromosome 10 was formed through NCF (Fig. 3e). It is likely that chromosome 1 and 11 were retained as independent chromosomes inherited from the AEK.

### Identification of *A. pictum* WRKYs

**Fig. 4.**
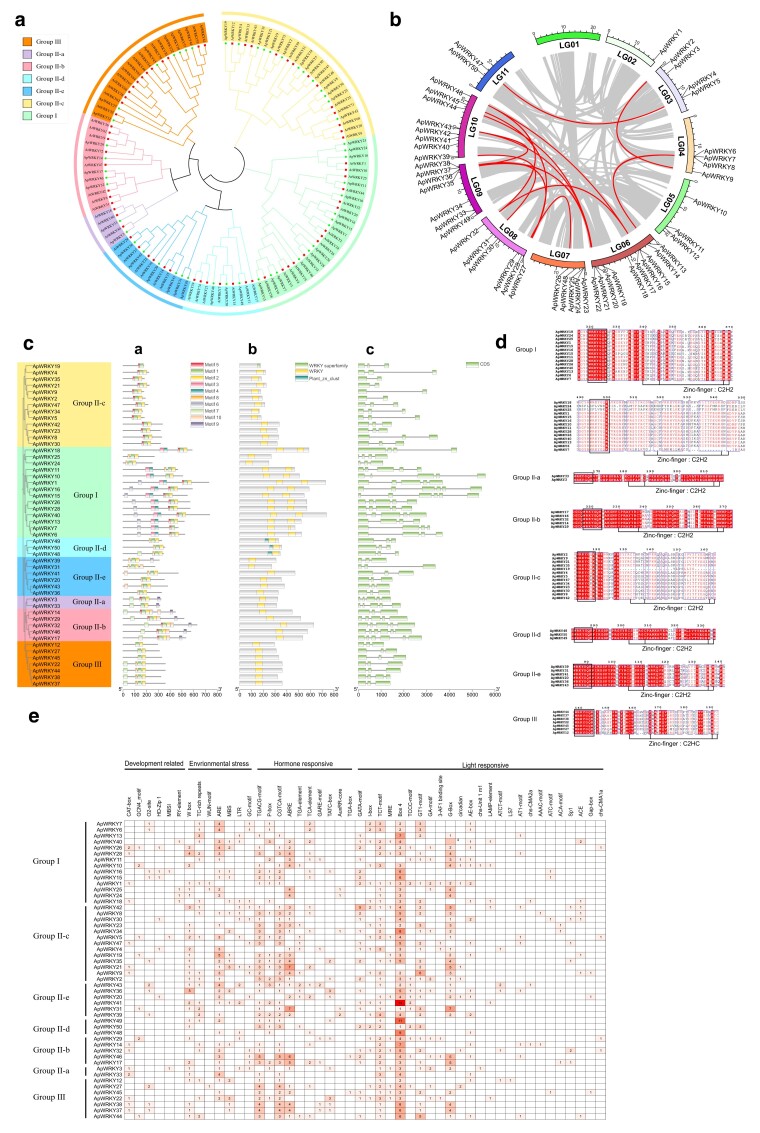
a) Neighbor-joining phylogenetic tree of WRKY proteins from *A. thaliana* and *A. pictum*. The WRKY proteins were classified into 3 groups (I–III), and group II proteins were classified into 5 subgroups (IIa–e). b) Distribution of *ApWRKY* genes and syntenic *ApWRKY* gene pairs on *A. pictum* chromosomes. c) Analysis of conserved protein motifs (a), conserved protein domains (b), and intron–exon structures of *ApWRKY* genes (c). Green boxes indicate exons. d) The conserved WRKY domain consists of the WRKYQK sequence followed by a zinc finger structure. e) Analysis of *cis*-acting elements in *ApWRKY* gene promoters. Cell color corresponds to the number of each element in the promoter of each *ApWRKY*.

We identified and characterized members of WRKY family in the drought-tolerant shrub *A. pictum*. Using *Arabidopsis* WRKY sequences from the TAIR (Berardini et al. 2015) database as queries, we identified 50 putative WRKY genes in the *A. pictum* genome (Fig. 4a). We first performed a comprehensive bioinformatic analysis of their predicted proteins (Supplementary Table 10), which ranged from 167 to 737 amino acids (aa) in length, with molecular weights from 19,193 to 80,662 Da and isoelectric points (pIs) from 4.90 to 9.76. All were classified as unstable proteins, and their aliphatic indices and GRAVY scores suggested a higher potential for hydrophilicity (Mohan and Venugopal 2012). Analysis of collinearity among the WRKY genes revealed 15 segmentally duplicated pairs across the *A. pictum* genome (Fig. 4b). Like the *Arabidopsis* WRKYs, the 50 ApWRKY proteins could be classified into 3 major groups (Eulgem et al. 2000): group I (2 WRKY domains and 2 C2H2 zinc finger motifs), group II (1 WRKY domain, 1 C2H2 zinc finger motif), and group III (1 WRKY domain, 1 C2HC zinc finger motif) (Fig. 4a). Group II could be divided into 5 subgroups (IIa–e), all of which contained *A. pictum* and *Arabidopsis* members.

Some of the *ApWRKYs* exhibited atypical variations in their conserved domains or zinc finger structures. The WRKY DNA-binding domain typically consists of the heptapeptide WRKYGQK (Xie et al. 2005), but this sequence was WRKYGKK in *ApWRKY5*, *47*, and *34*. The zinc finger motif was CX5C-HXH in *ApWRKY24*, *25*, and *10* (group I) and CX4C-HXH in *ApWRKY12* (group III) (Fig. 4d). Several *WRKYs* appeared to have lost specific domains. In group I, *ApWRKY24* and *ApWRKY25* contained only 1 WRKY domain and 1 zinc finger motif, and the N-terminal WRKY domain in *ApWRKY7* had lost the zinc finger motif. Similar loss of the zinc finger structure was observed in *ApWRKY19* from group IIc (Fig. 4d).

We next examined the conserved motifs and domains of the WRKY proteins and the intron–exon structures of the *WRKY* genes in the context of their phylogenetic relationships (Fig. 4c). All WRKY proteins contained at least 1 of the 2 major DNA-binding domains, which were composed of motifs 1/2 and 3/4, respectively, in the MEME analysis (Supplementary Fig. 1). In addition to the WRKY domain, the plant_zn_cluster domain was also found in *ApWRKY48*, *49*, and *50* from group IId. This domain is also present in group IId *WRKYs* from other drought-resistant species such as *Caragana korshinskii* and *Caragana intermedia* (Wan et al. 2018; Liu et al. 2023). The number of exons in *ApWRKY* genes ranged from 2 to 6, and genes from the same phylogenetic clade exhibited similar intron–exon structures (Fig. 4c). Moreover, a scanned was conducted on the 2,000 bp upstream of each *ApWRKY* transcription initiation site to detect potential *cis*-acting elements, and 48 elements were identified in the *WRKY* promoters (Fig. 4e).

### Expression profiles of *ApWRKYs* under drought stress

Leaf tissues of 4-week-old *A. pictum* seedlings were exposed to 30% PEG-induced drought stress for durations of 0, 4, and 12 h, with 3 replicates per time point. Subsequently, these samples were collected for transcriptome sequencing to explore alterations in the expression of *ApWRKY* genes in response to drought stress. Expression was quantified as TPM, and the log2(TPM + 1) values for each gene were hierarchically clustered and visualized in a heatmap (Supplementary Fig. 2).

**Fig. 5.**
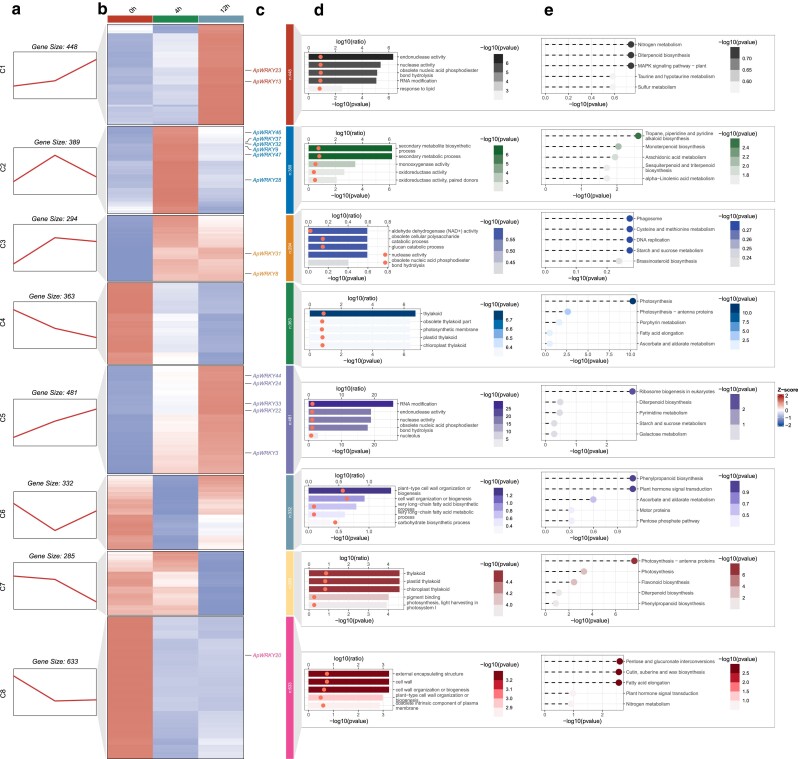
a) *DEGs exhibited* 8 temporal expression patterns over a 12-h PEG-mediated drought treatment. b) Clustered heatmap depicting DEG expression using TPM values with zero-mean normalization. c) *ApWRKY* genes that were differentially expressed under drought treatment. d and e) The top 5 significantly enriched GO terms d) and KEGG pathways e) of genes in each cluster.

A total of 3,225 DEGs were identified, including 16 *ApWRKY* genes, that were classified into 8 clusters (C1–C8) on the basis of their temporal expression patterns (Fig. 5a and c). Five *ApWRKYs* (*ApWRKY44*, *24*, *33*, *22*, and *3*) were present in C5, whose 481 members continued to increase in expression as the duration of drought stress increased. GO and KEGG enrichment analyses revealed that DEGs in this cluster were significantly enriched in RNA modification and ribosome biogenesis in eukaryotes (Fig. 5d and e). Notably, all drought-responsive *ApWRKYs* except *ApWRKY20* were found in 1 of the 4 upregulated gene clusters: C1 (2 *WRKYs*, 448 DEGs), C2 (6 *WRKYs*, 389 DEGs), C3 (2 *WRKYs*, 294 DEGs), and C5 (5 *WRKYs*, 481 DEGs). Members of these clusters were enriched in MAPK signaling pathway, secondary metabolic biosynthetic process, cysteine and methionine metabolism, and ribosome biogenesis in eukaryotes (Fig. 5d and e).

By contrast, *ApWRKY20* was present in C8, whose 633 members were downregulated with increasing drought duration and were significantly enriched in pentose and glucuronate interconversions, fatty acid elongation, cutin, and suberin and wax biosynthesis. Among the 3 smaller clusters of downregulated genes, C4 and C7 contained DEGs related to photosynthesis and thylakoids, consistent with declining photosynthetic activity under drought stress. C6 contained DEGs enriched in phenylpropanoid biosynthesis and plant hormone signal transduction whose expression first declined at 4 h and then increased again at 12 h.

## Discussion

Our study presents useful genomic and transcriptomic resources for further research on mechanisms of drought resistance in *A. pictum*, a drought-tolerant medicinal plant from the Tarim Basin, and offers insights into aspects of Apocynaceae evolution. The final assembled genome size was ∼225.3 Mb, with a scaffold N50 of 19.6 Mb and 11 pseudochromosomes. The high completeness, contiguity, correctness, and LAI score indicated that the *A. pictum* genome was of reference quality (Ou et al. 2018).

Significant variations in genome sizes are observed among the different subfamilies of Apocynaceae, as indicated by the available genome resources. The Rauvolfioideae subfamily is found to contain approximately 30,000 genes [30,085 protein-coding genes in *C. roseus* (Xu et al. 2023) and 33,300 in *V. thouarsii* (Cuello et al. 2022)], while the Apocynoideae subfamily [23,147 protein-coding genes in *A. pictum* and 21,327 in *A. venetum* (Dorjee et al. 2024)] and the Asclepiadoideae subfamily [21,899 in *M. tenacissima* (Zhou et al. 2023), 14,474 in *A. syriaca* (Weitemier et al. 2019b; Weitemier et al. 2019a), and 18,197 in *C. gigantea* (Hoopes et al. 2018)] consistently show around 20,000 genes. Interestingly, all 5 species in the Apocynoideae and Asclepiadoideae subfamilies have 11 chromosomes each. In contrast, the genomes of the species in Rauvolfioideae subfamily exhibit more complexity. For instance, *V. thouarsii* possesses the largest genome size (1,354.26 Mb), and *C. roseus* has the fewest chromosomes (8 chromosomes). These differences suggest that the Rauvolfioideae subfamily may have undergone more evolutionary events.

The family Apocynaceae has been classified into 5 subfamilies (Fishbein et al. 2018). To date, high-throughput genome sequencing has been performed on species from 3 of these subfamilies. A phylogenetic tree of selected Apocynaceae members based on high-throughput sequencing of nuclear genomic data revealed that Rauvolfioideae diverged first from the common ancestor of Rauvolfioideae, Apocynoideae, and Asclepiadoideae, consistent with previous research based on plastomes (Fishbein et al. 2018). As expected, the 2 representative Apocynoideae species, *A. pictum* and *A. venetum*, had the closest evolutionary relationship. Through the utilization of a larger genomic data set of the Apocynaceae family, we estimated the divergence time between *A. pictum* and *A. venetum* to be 2.2 MYA, which is earlier than previously estimated (Gao et al. 2023). The expanded genes of *A. pictum* were most highly enriched in the GO term “fatty acid metabolic process” (GO:0006631) and the KEGG pathway “protein processing in endoplasmic reticulum” (associated with 27 genes), which may be related to environmental stress responses in plants (Kachroo and Kachroo 2009; Liu and Howell 2010). The second most significantly enriched pathway was “flavonoid biosynthesis,” which could account for the differences in overall content and composition of flavonoids observed between *A. pictum* and *A. venetum* (Gao et al. 2021).

Previous studies have revealed that the AEK, which consists of 7 protochromosomes, experienced a whole-genome triplication event (WGT-γ) that resulted in the formation of the ACEK, with 21 chromosomes (Salse 2016). The syntenic depth revealed that the tested Apocynaceae species shared only the core eudicot WGT-γ event and had not undergone additional polyploidization events, similar to the model plant *V. vinifera* (Jaillon et al. 2007). In *A. pictum*, there has been limited rearrangement of 1, 4, and 11 chromosomes since the WGT-γ event, indicating that genes within these conserved chromosomes likely contribute to developmental processes and may have deterred chromosomal fusion. Karyotype assessments revealed that several chromosomes of *A. pictum* and *M. tenacissima* were derived completely from single AEK chromosomes, whereas *C. roseus* showed greater interchromosome rearrangement.

The WRKY TFs, named after their conserved N-terminal WRKY motifs, play crucial roles in various physiological processes, particularly in responses to abiotic stresses like drought, temperature, ultraviolet radiation, and salinity (Zhang and Wang 2005). In our study, 50 *ApWRKYs* were identified in *A. pictum*, and it was lower than *Arabidopsis* (over 70) but quite similar to the closely related species *C. roseus* (49) (Xu et al. 2023). We established their basic classifications and predicted the characteristics and conserved motifs of their encoded proteins. Some *ApWRKYs* had variations in their conserved domains. Previous work in rice showed that the WRKY family has been shaped by multiple episodes of domain acquisition and loss (Ross, Liu, and Shen 2007), and ApWRKY proteins identified here also appear to have experienced the loss of individual domains during evolution.

WRKY proteins bind to W-box elements in gene promoters to activate or inhibit transcription of downstream genes. They can also bind to their own promoters or to those of other *WRKYs* to create self-regulatory or cross-regulatory networks (Li et al. 2020). Based on the prediction of *cis*-acting elements, elements related to 5 plant hormones were identified: MeJA (TGACG-motif and CGTCA-motif), gibberellin (p-box, GARE-motif, and TATC-box), abscisic acid (ABRE), auxin (AuxRR-core, TGA-element, and TGA-box), and salicylic acid (TCA-element). *cis*-elements associated with stress-related hormones, such as the abscisic acid responsiveness element (ABRE), were particularly abundant in *ApWRKY* promoters. Seven stress-related *cis*-elements were also present, among which the W box, anaerobic induction element (ARE), and TC-rich repeats were particularly common. The abundance of ABA- and stress-related *cis*-elements in their promoters provides further evidence for the roles of *ApWRKYs* in regulating gene expression under abiotic stress.

As a desert plant, *A. pictum* exhibits high drought tolerance. A previous study has shown that PEG concentrations lower than 20% will not significantly affect the growth of *A. pictum* (Jiang et al. 2021). We subjected the seedlings to drought stress and conducted transcriptome sequencing. DESeq2 software was utilized to identify DEGs at each drought time point, while Mfuzz was used for soft clustering of the expression data. Sixteen *ApWRKY* genes showed changes in expression in response to PEG-mediated drought stress, with all except one showing upregulation. Differences in their expression patterns suggest that individual *ApWRKY* genes have distinct functions and may act at different stages of the drought response. For instance, *ApWRKYs* in cluster C1 were upregulated only at the later stage of PEG-mediated drought (12 h), those in C2 were upregulated only at the earlier stage (4 h), and those in C3 and C5 were upregulated at both stages. Most upregulated *ApWRKY* genes were in either cluster C2 or C5. To better understand their potential functions, we investigated the roles of their *Arabidopsis* homologs from the phylogenetic tree. *AtWRKY33*, the *Arabidopsis* homolog of *ApWRKY28*, directly and negatively regulates the cellulose synthase gene *CesA8*, resulting in improved tolerance to drought stress (Wang et al. 2013). *ApWRKY28*, whose gene was upregulated at 4 h, may have a similar function. *ApWRKY47* is homologous to *AtWRKY50* from *Arabidopsis* and *HcWRKY50* from kenaf (*Hibiscus cannabinus*), and all 3 proteins contain the variant WRKYGKK motif rather than the common WRKYGQK motif. A previous study demonstrated that *HcWRKY50* positively regulates expression of *RD29B* and *COR47* in the ABA signaling pathway (Niu et al. 2022), suggesting that *ApWRKY47* may function similarly in ABA signaling and drought tolerance. Overexpression of the *ApWRKY22* homolog *AtWRKY30* improved heat and drought tolerance in wheat (El-Esawi et al. 2019). The *ApWRKY33* homologs *AtWRKY18*/*60* and the *ApWRKY3* homolog *AtWRKY40* form an interconnected regulatory network in *Arabidopsis* that controls the expression of genes associated with ABA, defense, and stress responses, in which *AtWRKY18*/*60* act as transcriptional activators and *AtWRKY40* as a repressor (Chen et al. 2010). The roles of their *Arabidopsis* homologs in drought and stress tolerance suggest that *ApWRKY28*, *47*, *22*, *33*, and *3* are good candidates for future research on mechanisms of *A. pictum* responses to abiotic stress.

In summary, the availability of a high-quality genome provides new genetic resources for future evolutionary and comparative genomics analyses of *A. pictum* and other Apocynaceae species, and transcriptomic data provide initial molecular insights into drought adaptation in this desert plant.

## Supplementary Material

jkae237_Supplementary_Data

## Data Availability

All raw sequence reads produced in this study, including Illumina, Nanopore, and Hi-C interaction reads and RNA-seq data from drought treatments, have been deposited at the NCBI Sequence Read Archive (SRA) under BioProject PRJNA1069313. Custom scripts and pipeline used throughout are available on GitHub (https://github.com/Phantom-Aria/Ap_genome_assembly). Pertinent data sets, comprising the final assembled genome and its annotations, have been deposited at Figshare with the DOI 10.6084/m9.figshare.25060931. Supplemental material available at G3 online.

## References

[jkae237-B1] Benson G . 1999. Tandem repeats finder: a program to analyze DNA sequences. Nucleic Acids Res. 27(2):573–580. doi:10.1093/nar/27.2.573. 9862982 PMC148217

[jkae237-B2] Berardini TZ , Reiser L, Li D, Mezheritsky Y, Muller R, Strait E, Huala E. 2015. The Arabidopsis information resource: making and mining the “gold standard” annotated reference plant genome. Genesis. 53(8):474–485. doi:10.1002/dvg.22877. 26201819 PMC4545719

[jkae237-B3] Bruna T , Lomsadze A, Borodovsky M. 2020. GeneMark-EP+: eukaryotic gene prediction with self-training in the space of genes and proteins. NAR Genom Bioinform. 2(2):lqaa026. doi:10.1093/nargab/lqaa026. 32440658 PMC7222226

[jkae237-B4] Buchfink B , Reuter K, Drost H-G. 2021. Sensitive protein alignments at tree-of-life scale using DIAMOND. Nat Methods. 18(4):366–368. doi:10.1038/s41592-021-01101-x. 33828273 PMC8026399

[jkae237-B5] Camacho C , Coulouris G, Avagyan V, Ma N, Papadopoulos J, Bealer K, Madden TL. 2009. BLAST+: architecture and applications. BMC Bioinformatics. 10:421. doi:10.1186/1471-2105-10-421. 20003500 PMC2803857

[jkae237-B6] Chen C , Chen H, Zhang Y, Thomas HR, Frank MH, He Y, Xia R. 2020. TBtools: an integrative toolkit developed for interactive analyses of big biological data. Mol Plant. 13(8):1194–1202. doi:10.1016/j.molp.2020.06.009. 32585190

[jkae237-B7] Chen H , Lai Z, Shi J, Xiao Y, Chen Z, Xu X. 2010. Roles of Arabidopsis WRKY18, WRKY40 and WRKY60 transcription factors in plant responses to abscisic acid and abiotic stress. BMC Plant Biol. 10(1):281. doi:10.1186/1471-2229-10-281. 21167067 PMC3023790

[jkae237-B8] Chen S , Zhou Y, Chen Y, Gu J. 2018. Fastp: an ultra-fast all-in-one FASTQ preprocessor. Bioinformatics. 34(17):i884–ii90. doi:10.1093/bioinformatics/bty560. 30423086 PMC6129281

[jkae237-B9] Cuello C , Stander EA, Jansen HJ, Dugé de Bernonville T, Lanoue A, Giglioli-Guivarc'h N, Papon N, Dirks RP, Jensen MK, O'Connor SE, et al 2022. Genome assembly of the medicinal plant *Voacanga thouarsii*. Genome Biol Evol. 14(11):evac158. doi:10.1093/gbe/evac158. 36300641 PMC9673491

[jkae237-B10] Del Rio C , Wang T-X, Liu J, Liang S-Q, Spicer RA, Wu F-X, Zhou Z-K, Su T. 2020. Asclepiadospermum gen. nov., the earliest fossil record of Asclepiadoideae (Apocynaceae) from the early Eocene of central Qinghai-Tibetan Plateau, and its biogeographic implications. Am J Bot. 107(1):126–138. doi:10.1002/ajb2.1418. 31944266

[jkae237-B11] Dobin A , Davis CA, Schlesinger F, Drenkow J, Zaleski C, Jha S, Batut P, Chaisson M, Gingeras TR. 2013. STAR: ultrafast universal RNA-seq aligner. Bioinformatics. 29(1):15–21. doi:10.1093/bioinformatics/bts635. 23104886 PMC3530905

[jkae237-B12] Dorjee T , Tan J, Zuo Q, Zheng L, Liu Q, Sun H, Zhou Y, Gao F. 2024. Chromosome-scale genome analysis of *Apocynum venetum* sheds light on *Apocynum* phylogenetics, bast fiber development, and flavonoid synthesis. Ind Crops Prod. 212:118325. doi:10.1016/j.indcrop.2024.118325.

[jkae237-B13] Dudchenko O , Batra SS, Omer AD, Nyquist SK, Hoeger M, Durand NC, Shamim MS, Machol I, Lander ES, Presser Aiden A, et al 2017. De novo assembly of the *Aedes aegypti* genome using Hi-C yields chromosome-length scaffolds. Science (1979). 356(6333):92–95. doi:10.1126/science.aal3327. PMC5635820 28336562

[jkae237-B14] Durand NC , Robinson JT, Shamim MS, Machol I, Mesirov JP, Lander ES, Aiden EL. 2016. Juicebox provides a visualization system for Hi-C contact maps with unlimited zoom. Cell Syst. 3(1):99–101. doi:10.1016/j.cels.2015.07.012. 27467250 PMC5596920

[jkae237-B15] El-Esawi MA , Al-Ghamdi AA, Ali HM, Ahmad M. 2019. Overexpression of AtWRKY30 transcription factor enhances heat and drought stress tolerance in wheat (Triticum aestivum L.). Genes (Basel). 10(2):163. doi:10.3390/genes10020163. 30791662 PMC6410048

[jkae237-B16] Ellinghaus D , Kurtz S, Willhoeft U. 2008. LTRharvest, an efficient and flexible software for de novo detection of LTR retrotransposons. BMC Bioinformatics. 9(1):18. doi:10.1186/1471-2105-9-18. 18194517 PMC2253517

[jkae237-B17] Emms DM , Kelly S. 2018. STAG: species tree inference from all genes. [preprint] bioRxiv: 267914. doi:10.1101/267914.

[jkae237-B18] Emms DM , Kelly S. 2019. OrthoFinder: phylogenetic orthology inference for comparative genomics. Genome Biol. 20(1):238. doi:10.1186/s13059-019-1832-y. 31727128 PMC6857279

[jkae237-B19] Eulgem T , Rushton PJ, Robatzek S, Somssich IE. 2000. The WRKY superfamily of plant transcription factors. Trends Plant Sci. 5(5):199–206. doi:10.1016/S1360-1385(00)01600-9. 10785665

[jkae237-B20] Fishbein M , Livshultz T, Straub SC, Simões AO, Boutte J, McDonnell A, Foote A. 2018. Evolution on the backbone: Apocynaceae phylogenomics and new perspectives on growth forms, flowers, and fruits. Am J Bot. 105(3):495–513. doi:10.1002/ajb2.1067. 29733432

[jkae237-B21] Flynn JM , Hubley R, Goubert C, Rosen J, Clark AG, Feschotte C, Smit AF. 2020. RepeatModeler2 for automated genomic discovery of transposable element families. Proc Natl Acad Sci U S A. 117(17):9451–9457. doi:10.1073/pnas.1921046117. 32300014 PMC7196820

[jkae237-B22] Gabriel L , Bruna T, Hoff KJ, Ebel M, Lomsadze A, Borodovsky M, Stanke M. 2024. BRAKER3: fully automated genome annotation using RNA-Seq and protein evidence with GeneMark-ETP, AUGUSTUS and TSEBRA. [preprint] Genome Res. doi:10.1101/gr.278090.123. PMC11216308 38866550

[jkae237-B23] Gao G , Abubakar AS, Chen J, Chen P, Chen K, Yu C, Wang X, Qiu X, Huang X, Shao D, et al 2023. Comparative genome and metabolome analyses uncover the evolution and flavonoid biosynthesis between *Apocynum venetum* and *Apocynum hendersonii*. iScience. 26(5):106772. doi:10.1016/j.isci.2023.106772. 37250304 PMC10214733

[jkae237-B24] Gao G , Liu N, Yu C, Chen P, Chen J, Chen K, Wang X, Liu B, Zhu A. 2021. UPLC-ESI-MS/MS based characterization of active flavonoids from *Apocynum* spp. and anti-bacteria assay. Antioxidants. 10(12):1901. doi:10.3390/antiox10121901. 34943004 PMC8750526

[jkae237-B25] Gurevich A , Saveliev V, Vyahhi N, Tesler G. 2013. QUAST: quality assessment tool for genome assemblies. Bioinformatics. 29(8):1072–1075. doi:10.1093/bioinformatics/btt086. 23422339 PMC3624806

[jkae237-B26] Hoff KJ , Lomsadze A, Borodovsky M, Stanke M. 2019. Whole-genome annotation with BRAKER. Methods Mol Biol. 1962:65–95. doi:10.1007/978-1-4939-9173-0_5. 31020555 PMC6635606

[jkae237-B27] Hoopes GM , Hamilton JP, Kim J, Zhao D, Wiegert-Rininger K, Crisovan E, Robin Buell C. 2018. Genome assembly and annotation of the medicinal plant *Calotropis gigantea*, a producer of anticancer and antimalarial cardenolides’. G3 (Bethesda). 8(2):385–391. doi:10.1534/g3.117.300331. 29237703 PMC5919723

[jkae237-B28] Hu J , Fan J, Sun Z, Liu S. 2020. NextPolish: a fast and efficient genome polishing tool for long-read assembly. Bioinformatics. 36(7):2253–2255. doi:10.1093/bioinformatics/btz891. 31778144

[jkae237-B29] Hu J , Wang Z, Sun Z, Hu B, Oluwakemi Ayoola A, Liang F, Li J, Sandoval JR, Cooper DN, Ye K, et al 2024. NextDenovo: an efficient error correction and accurate assembly tool for noisy long reads. Genome Biol. 25(1):107. doi:10.1186/s13059-024-03252-4. 38671502 PMC11046930

[jkae237-B30] Huerta-Cepas J , Szklarczyk D, Heller D, Hernández-Plaza A, Forslund SK, Cook H, Mende DR, Letunic I, Rattei T, Jensen LJ, et al 2019. eggNOG 5.0: a hierarchical, functionally and phylogenetically annotated orthology resource based on 5090 organisms and 2502 viruses. Nucleic Acids Res. 47(D1):D309–DD14. doi:10.1093/nar/gky1085. 30418610 PMC6324079

[jkae237-B31] Jaillon O , Aury JM, Noel B, Policriti A, Clepet C, Casagrande A, Choisne N, Aubourg S, Vitulo N, Jubin C. et al 2007. ‘The grapevine genome sequence suggests ancestral hexaploidization in major angiosperm phyla’. Nature. 449(7161):463–467. doi:10.1038/nature06148. 17721507

[jkae237-B32] Jiang L , She C, Tian C, Tanveer M, Wang L. 2021. Storage period and different abiotic factors regulate seed germination of two apocynum species—cash crops in arid saline regions in the Northwestern China. Front Plant Sci. 12:671157. doi:10.3389/fpls.2021.671157. 34220893 PMC8248540

[jkae237-B33] Jiang N , Zhang Q, Zhang S, Zhao X, Cheng H. 2022. Spatial and temporal evolutions of vegetation coverage in the Tarim River Basin and their responses to phenology. Catena (Amst). 217:106489. doi:10.1016/j.catena.2022.106489.

[jkae237-B34] Jurka J , Kapitonov VV, Pavlicek A, Klonowski P, Kohany O, Walichiewicz J. 2005. Repbase update, a database of eukaryotic repetitive elements. Cytogenet Genome Res. 110(1-4):462–467. doi:10.1159/000084979. 16093699

[jkae237-B35] Kachroo A , Kachroo P. 2009. Fatty acid–derived signals in plant defense. Annu Rev Phytopathol. 47(1):153–176. doi:10.1146/annurev-phyto-080508-081820. 19400642

[jkae237-B36] Kalvari I , Nawrocki EP, Ontiveros-Palacios N, Argasinska J, Lamkiewicz K, Marz M, Griffiths-Jones S, Toffano-Nioche C, Gautheret D, Weinberg Z, et al 2020. Rfam 14: expanded coverage of metagenomic, viral and microRNA families. Nucleic Acids Res. 49(D1):D192–D200. doi:10.1093/nar/gkaa1047. PMC7779021 33211869

[jkae237-B37] Katoh K , Standley DM. 2013. MAFFT multiple sequence alignment software version 7: improvements in performance and usability. Mol Biol Evol. 30(4):772–780. doi:10.1093/molbev/mst010. 23329690 PMC3603318

[jkae237-B38] Kumar L , Futschik ME. 2007. Mfuzz: a software package for soft clustering of microarray data. Bioinformation. 2(1):5–7. doi:10.6026/97320630002005. 18084642 PMC2139991

[jkae237-B39] Kumar S , Stecher G, Li M, Knyaz C, Tamura K. 2018. MEGA x: molecular evolutionary genetics analysis across computing platforms. Mol Biol Evol. 35(6):1547–1549. doi:10.1093/molbev/msy096. 29722887 PMC5967553

[jkae237-B40] Kumar S , Stecher G, Suleski M, Hedges SB. 2017. TimeTree: a resource for timelines, timetrees, and divergence times’. Mol Biol Evol. 34(7):1812–1819. doi:10.1093/molbev/msx116. 28387841

[jkae237-B41] Kuznetsov D , Tegenfeldt F, Manni M, Seppey M, Berkeley M, Kriventseva EV, Zdobnov EM. 2023. OrthoDB v11: annotation of orthologs in the widest sampling of organismal diversity. Nucleic Acids Res. 51(D1):D445–DD51. doi:10.1093/nar/gkac998. 36350662 PMC9825584

[jkae237-B42] Lescot M , Déhais P, Thijs G, Marchal K, Moreau Y, Van de Peer Y, Rouzé P, Rombauts S. 2002. PlantCARE, a database of plant cis-acting regulatory elements and a portal to tools for in silico analysis of promoter sequences. Nucleic Acids Res. 30(1):325–327. doi:10.1093/nar/30.1.325. 11752327 PMC99092

[jkae237-B43] Li W , Pang S, Lu Z, Jin B. 2020. Function and mechanism of WRKY transcription factors in abiotic stress responses of plants. Plants (Basel). 9(11):1515. doi:10.3390/plants9111515. 33171689 PMC7695288

[jkae237-B44] Liu J-X , Howell SH. 2010. Endoplasmic reticulum protein quality control and its relationship to environmental stress responses in plants. Plant Cell. 22(9):2930–2942. doi:10.1105/tpc.110.078154. 20876830 PMC2965551

[jkae237-B45] Liu J , Li G, Wang R, Wang G, Wan Y. 2023. Genome-wide analysis of WRKY transcription factors involved in abiotic stress and ABA response in *Caragana korshinskii*. Int J Mol Sci. 24(11):9519. doi:10.3390/ijms24119519. 37298467 PMC10253768

[jkae237-B46] Ma M , Hong C-L, An S-Q, Li B. 2003. Seasonal, spatial, and interspecific variation in quercetin in *Apocynum venetum* and *Poacynum hendersonii*, Chinese traditional herbal teas. J Agric Food Chem. 51(8):2390–2393. doi:10.1021/jf021055i. 12670186

[jkae237-B47] McKenna A , Hanna M, Banks E, Sivachenko A, Cibulskis K, Kernytsky A, Garimella K, Altshuler D, Gabriel S, Daly M, et al 2010. The genome analysis toolkit: a MapReduce framework for analyzing next-generation DNA sequencing data. Genome Res. 20(9):1297–1303. doi:10.1101/gr.107524.110. 20644199 PMC2928508

[jkae237-B48] Mendes FK , Vanderpool D, Fulton B, Hahn MW. 2020. CAFE 5 models variation in evolutionary rates among gene families. Bioinformatics. 36(22-23):5516–5518. doi:10.1093/bioinformatics/btaa1022. 33325502

[jkae237-B49] Mistry J , Chuguransky S, Williams L, Qureshi M, Salazar GA, Sonnhammer EL, Tosatto SC, Paladin L, Raj S, Richardson LJ, et al 2021. Pfam: the protein families database in 2021. Nucleic Acids Res. 49(D1):D412–DD19. doi:10.1093/nar/gkaa913. 33125078 PMC7779014

[jkae237-B50] Mohan R , Venugopal S. 2012. Computational structural and functional analysis of hypothetical proteins of *Staphylococcus aureus*. Bioinformation. 8(15):722–728. doi:10.6026/97320630008722. 23055618 PMC3449381

[jkae237-B51] Nawrocki EP , Eddy SR. 2013. ‘Infernal 1.1: 100-fold faster RNA homology searches’. Bioinformatics. 29(22):2933–2935. doi:10.1093/bioinformatics/btt509. 24008419 PMC3810854

[jkae237-B52] Niu X , Chen M, She Z, Aslam M, Qi J, Qin Y. 2022. Ectopic expression of kenaf (*Hibiscus cannabinus* L.) HcWRKY50 improves plants’ tolerance to drought stress and regulates ABA signaling in Arabidopsis. Agronomy. 12(5):1176. doi:10.3390/agronomy12051176.

[jkae237-B53] O'Connor BD , van der Auwera G. 2020. Genomics in the cloud: using Docker, GATK, and WDL in Terra. Beijing: O'Reilly Media, Incorporated.

[jkae237-B54] Ou S , Chen J, Jiang N. 2018. Assessing genome assembly quality using the LTR assembly Index (LAI). Nucleic Acids Res. 46(21):e126. doi:10.1093/nar/gky730. 30107434 PMC6265445

[jkae237-B55] Ou S , Jiang N. 2018. LTR_retriever: a highly accurate and sensitive program for identification of long terminal repeat retrotransposons. Plant Physiol. 176(2):1410–1422. doi:10.1104/pp.17.01310. 29233850 PMC5813529

[jkae237-B56] Ranallo-Benavidez TR , Jaron KS, Schatz MC. 2020. GenomeScope 2.0 and Smudgeplot for reference-free profiling of polyploid genomes. Nat Commun. 11(1):1432. doi:10.1038/s41467-020-14998-3. 32188846 PMC7080791

[jkae237-B57] Reis Md , Yang Z. 2011. Approximate likelihood calculation on a phylogeny for Bayesian estimation of divergence times. Mol Biol Evol. 28(7):2161–2172. doi:10.1093/molbev/msr045. 21310946

[jkae237-B58] Ribeiro PL , Rapini A, Damascena LS, van den Berg C. 2014. Plant diversification in the Espinhaço Range: insights from the biogeography of Minaria (Apocynaceae). Taxon. 63(6):1253–1264. doi:10.12705/636.16.

[jkae237-B59] Ross CA , Liu Y, Shen QJ. 2007. The WRKY gene family in rice (*Oryza sativa*). J Integr Plant Biol. 49(6):827–842. doi:10.1111/j.1744-7909.2007.00504.x.

[jkae237-B60] Rouzi A , Halik Ü, Thevs N, Welp M, Aishan T. 2017. Water efficient alternative crops for sustainable agriculture along the Tarim basin: a comparison of the economic potentials of *Apocynum pictum*, Chinese red date and cotton in Xinjiang, China. Sustainability. 10(1):35. doi:10.3390/su10010035.

[jkae237-B61] Salse J . 2016. Ancestors of modern plant crops. Curr Opin Plant Biol. 30:134–142. doi:10.1016/j.pbi.2016.02.005. 26985732

[jkae237-B62] Simão FA , Waterhouse RM, Ioannidis P, Kriventseva EV, Zdobnov EM. 2015. BUSCO: assessing genome assembly and annotation completeness with single-copy orthologs. Bioinformatics. 31(19):3210–3212. doi:10.1093/bioinformatics/btv351. 26059717

[jkae237-B63] Stanke M , Keller O, Gunduz I, Hayes A, Waack S, Morgenstern B. 2006. AUGUSTUS: ab initio prediction of alternative transcripts. Nucleic Acids Res. 34(Web Server):W435–WW39. doi:10.1093/nar/gkl200. 16845043 PMC1538822

[jkae237-B64] Storer J , Hubley R, Rosen J, Wheeler TJ, Smit AF. 2021. The Dfam community resource of transposable element families, sequence models, and genome annotations. Mob DNA. 12(1):2. doi:10.1186/s13100-020-00230-y. 33436076 PMC7805219

[jkae237-B65] Sun P , Jiao B, Yang Y, Shan L, Li T, Li X, Xi Z, Wang X, Liu J. 2022. WGDI: a user-friendly toolkit for evolutionary analyses of whole-genome duplications and ancestral karyotypes. Mol Plant. 15(12):1841–1851. doi:10.1016/j.molp.2022.10.018. 36307977

[jkae237-B66] Sun J , Zhang L, Deng C, Zhu R. 2008. Evidence for enhanced aridity in the Tarim basin of China since 5.3 ma. Quat Sci Rev. 27(9-10):1012–1023. doi:10.1016/j.quascirev.2008.01.011.

[jkae237-B67] Wan Y , Mao M, Wan D, Yang Q, Yang F, Mandlaa GL, Wang R. 2018. Identification of the WRKY gene family and functional analysis of two genes in *Caragana intermedia*. BMC Plant Biol. 18(1):31. doi:10.1186/s12870-018-1235-3. 29426284 PMC5807834

[jkae237-B68] Wang J , Chitsaz F, Derbyshire MK, Gonzales NR, Gwadz M, Lu S, Marchler GH, Song JS, Thanki N, Yamashita RA, et al 2023. The conserved domain database in 2023. Nucleic Acids Res. 51(D1):D384–DD88. doi:10.1093/nar/gkac1096. 36477806 PMC9825596

[jkae237-B69] Wang X , Du B, Liu M, Sun N, Qi X. 2013. Arabidopsis transcription factor WRKY33 is involved in drought by directly regulating the expression of CesA8. Am J Plant Sci. 4(6A):21–27. doi:10.4236/ajps.2013.46A004.

[jkae237-B70] Wang Z , Li Y, Sun P, Zhu M, Wang D, Lu Z, Hu H, Xu R, Zhang J, Ma J, et al 2022. A high-quality Buxus austro-yunnanensis (Buxales) genome provides new insights into karyotype evolution in early eudicots. BMC Biol. 20(1):216. doi:10.1186/s12915-022-01420-1. 36195948 PMC9533543

[jkae237-B71] Wang Y , Tang H, DeBarry JD, Tan X, Li J, Wang X, Lee T-h, Jin H, Marler B, Guo H. 2012. MCScanX: a toolkit for detection and evolutionary analysis of gene synteny and collinearity. Nucleic Acids Res. 40(7):e49. doi:10.1093/nar/gkr1293. 22217600 PMC3326336

[jkae237-B72] Weitemier K , Straub SCK, Fishbein M, Bailey CD, Cronn RC, Liston A. 2019. A draft genome and transcriptome of common milkweed (*Asclepias syriaca*) as resources for evolutionary, ecological, and molecular studies in milkweeds and Apocynaceae. PeerJ. 7:e7649. doi:10.7717/peerj.7649. 31579586 PMC6756140

[jkae237-B73] Wu T , Hu E, Xu S, Chen M, Guo P, Dai Z, Feng T, Zhou L, Tang W, Zhan L, et al 2021. ‘clusterProfiler 4.0: a universal enrichment tool for interpreting omics data’. The innovation. 2(3):100141. doi:10.1016/j.xinn.2021.100141. 34557778 PMC8454663

[jkae237-B74] Xie J , Chen Y, Cai G, Cai R, Hu Z, Wang H. 2023. Tree visualization by one table (tvBOT): a web application for visualizing, modifying and annotating phylogenetic trees. Nucleic Acids Res. 51(W1):W587–WW92. doi:10.1093/nar/gkad359. 37144476 PMC10320113

[jkae237-B75] Xie W , Zhang X, Wang T, Hu J. 2012. Botany, traditional uses, phytochemistry and pharmacology of *Apocynum venetum* L.(Luobuma): a review. J Ethnopharmacol. 141(1):1–8. doi:10.1016/j.jep.2012.02.003. 22421379

[jkae237-B76] Xie Z , Zhang Z-L, Zou X, Huang J, Ruas P, Thompson D, Shen QJ. 2005. Annotations and functional analyses of the rice WRKY gene superfamily reveal positive and negative regulators of abscisic acid signaling in aleurone cells. Plant Physiol. 137(1):176–189. doi:10.1104/pp.104.054312. 15618416 PMC548849

[jkae237-B77] Xu Z , Wang H. 2007. LTR_FINDER: an efficient tool for the prediction of full-length LTR retrotransposons. Nucleic Acids Res. 35(Web Server issue):W265–WW68. doi:10.1093/nar/gkm286. 17485477 PMC1933203

[jkae237-B78] Xu Z , Wang G, Wang Q, Li X, Zhang G, Qurban A, Zhang C, Zhou Y, Si H, Hu L, et al 2023. A near-complete genome assembly of *Catharanthus roseus* and insights into its vinblastine biosynthesis and high susceptibility to the Huanglongbing pathogen. Plant Commun. 4(6):100661. doi:10.1016/j.xplc.2023.100661. 37464741 PMC10721464

[jkae237-B79] Yang Z . 2007. PAML 4: phylogenetic analysis by maximum likelihood. Mol Biol Evol. 24(8):1586–1591. doi:10.1093/molbev/msm088. 17483113

[jkae237-B80] Yang J , Zhang L, Jiang L, Zhan YG, Fan GZ. 2021. Quercetin alleviates seed germination and growth inhibition in *Apocynum venetum* and *Apocynum pictum* under mannitol-induced osmotic stress. Plant Physiol Biochem. 159:268–276. doi:10.1016/j.plaphy.2020.12.025. 33401201

[jkae237-B81] Zhang R-G , Li G-Y, Wang X-L, Dainat J, Wang Z-X, Ou S, Ma Y. 2022. TEsorter: an accurate and fast method to classify LTR-retrotransposons in plant genomes. Hortic Res. 9:uhac017. doi:10.1093/hr/uhac017. 35184178 PMC9002660

[jkae237-B82] Zhang Y , Wang L. 2005. The WRKY transcription factor superfamily: its origin in eukaryotes and expansion in plants. BMC Evol Biol. 5:1. doi:10.1186/1471-2148-5-1. 15629062 PMC544883

[jkae237-B83] Zheng C , Fan J, Caraballo-Ortiz MA, Liu Y, Liu T, Fu G, Zhang Y, Yang P, Su X. 2022. The complete chloroplast genome and phylogenetic relationship of *Apocynum pictum* (Apocynaceae), a Central Asian shrub and second-class national protected species of western China. Gene. 830:146517. doi:10.1016/j.gene.2022.146517. 35452705

[jkae237-B84] Zhou Y , Fan W, Zhang H, Zhang J, Zhang G, Wang D, Xiang G, Zhao C, Li L, He S, et al 2023. *Marsdenia tenacissima* genome reveals calcium adaptation and tenacissoside biosynthesis. Plant J. 113(6):1146–1159. doi:10.1111/tpj.16081. 36575579

