# Supplementary material for: Chromosome-scale genome assembly of Apocynum pictum, a drought-tolerant medicinal plant from the Tarim Basin
Source: G3 (Bethesda). 2024 Nov 5;14(12):jkae237. doi: 10.1093/g3journal/jkae237 (PMC11631493; doi:10.1093/g3journal/jkae237)
Supplement: jkae237_Supplementary_Data [file jkae237_supplementary_data.docx]

**Tables**

**Supplementary Table S1. DNA sequencing data for *A. pictum* genome assembly.**

| **Read type** | **Number of clean reads** | **Clean bases (bp)** |
| --- | --- | --- |
| **Illumina reads** | 114,723,423 | 16,987,165,200 |
| **Hi-C reads** | 331,704,418 | 48,263,673,286 |
| **ONT reads** | 1,870,453 | 21,486,292,331 |

**Supplementary Table S2. BUSCO results for *A. pictum* genome assembly and annotation.**

| **Class of BUSCO** | **Assembly** | **Annotation** |
| --- | --- | --- |
| **Complete BUSCOs (C)** | 2250 (96.8%) | 2232 (96%) |
| **Complete and single-copy BUSCOs (S)** | 2225 (95.7%) | 1837 (79.0%) |
| **Complete and duplicated BUSCOs (D)** | 25 (1.1%) | 395 (17.0%) |
| **Fragmented BUSCOs (F)** | 23 (1.0%) | 6 (0.3%) |
| **Missing BUSCOs (M)** | 53 (2.2%) | 88 (3.7%) |
| **Total eudicot BUSCOs** | 2326 | 2326 |

**Supplementary Table S3. Genome base-accuracy statistics.**

| **Homozygous SNPs** | **Homozygous indels** | **Bases (bp)** | **Error rate based on SNPs (%)** | **Genome accuracy (%)** |
| --- | --- | --- | --- | --- |
| 9033 | 15,965 | 225,320,346 | 0.01109 | 99.98891 |

**Supplementary Table S4. TRF top-15 tandem repeat statistics.**

| **Motif (-mer)** | **Number** | **Length (bp)** | **Percentage (%)** |
| --- | --- | --- | --- |
| **21** | 2454 | 124,223 | 4.45 |
| **23** | 2172 | 124,981 | 3.94 |
| **22** | 1816 | 106,371 | 3.29 |
| **20** | 1574 | 72,179 | 2.86 |
| **18** | 1573 | 75,461 | 2.85 |
| **15** | 1459 | 55,329 | 2.65 |
| **14** | 1368 | 57,538 | 2.48 |
| **17** | 1341 | 54,261 | 2.43 |
| **13** | 1339 | 49,732 | 2.43 |
| **16** | 1333 | 53,619 | 2.42 |
| **3** | 1253 | 60,014 | 2.27 |
| **24** | 1213 | 89,714 | 2.20 |
| **19** | 1169 | 52,447 | 2.12 |
| **12** | 1098 | 48,310 | 1.99 |
| **32** | 1002 | 103,134 | 1.82 |
| **Other** | 32,967 | 4,818,015 | 59.80 |
| **Total** | 55,131 | 5,945,328 | 100.00 |

**Supplementary Table S5. Classification of repetitive elements in the *A. pictum* genome.**

| **Class** | **Order** | **Superfamily** | **Number of elements** | **Length (bp)** | **Percentage (%)** |
| --- | --- | --- | --- | --- | --- |
| Class I | LINEs | L1/CIN4 | 4542 | 3,199,382 | 1.42 |
|  |  | RTE/Bov-B | 92 | 17,646 | 0.01 |
|  | LTR retrotransposons | Copia | 18,033 | 19,585,830 | 8.69 |
|  |  | Gypsy | 20,563 | 22,441,741 | 9.96 |
| Class II | DNA transposons | hobo-Activator | 8911 | 3,376,463 | 1.50 |
|  |  | Tc1-IS630-Pogo | 6451 | 1,126,984 | 0.50 |
|  |  | Tourist/Harbinger | 2002 | 616,188 | 0.27 |
| Rolling-circles |  |  | 710 | 255,029 | 0.11 |
| Tandem Repeats |  |  | 55,131 | 5,945,328 | 2.64 |
| Unclassified |  |  | 108,008 | 24,431,790 | 10.84 |
| Total Repeats |  |  | 224,443 | 80,996,381 | 35.95 |

**Supplementary Table S6. ncRNAs in the *A. pictum* genome.**

| **ncRNA type** | **Number of elements** | **Total length (bp)** |
| --- | --- | --- |
| **miRNA** | 85 | 11,530 |
| **snRNA** | 549 | 59,195 |
| **tRNA** | 416 | 30,750 |
| **rRNA** | 688 | 637,008 |
| **Others** | 89 | 9460 |
| **Total** | 1827 | 747,943 |

**Supplementary Table S7. Summary of protein-coding gene predictions.**

| **Gene set** | **Total genes predicted** | **Average gene length (bp)** | **Average CDS length (bp)** | **Average exon length (bp)** | **Average intron length (bp)** |
| --- | --- | --- | --- | --- | --- |
| **RNA seq** | 12,603 | 4258 | 1357 | 182 | 512 |
| **Homolog** | 11,460 | 4368 | 1386 | 211 | 534 |
| ***De novo*** | 23,047 | 3300 | 1258 | 229 | 502 |
| **Final Set** | 23,147 | 3574 | 1303 | 233 | 539 |

**Supplementary Table S8. Functional annotations of *A. pictum* genes.**

| **Database** | **Number of genes** | **Percentage (%)** |
| --- | --- | --- |
| **Pfam** | 18,821 | 81.31 |
| **KEGG** | 10,558 | 45.61 |
| **COG** | 19,553 | 84.47 |
| **GO** | 11,063 | 47.79 |
| **eggNOG** | 21,148 | 91.36 |
| **Total** | 23,147 | 100 |

**Supplementary Table S9. Summary of gene family clustering.**

| **Species** | **Total genes** | **Families** | **Genes in families** | **Unique genes** | **Unclustered genes** |
| --- | --- | --- | --- | --- | --- |
| ***O. sativa*** | 41,961 | 14,299 | 33,542 | 13,629 | 8,419 |
| ***A. thaliana*** | 27,337 | 13,344 | 25,052 | 4,299 | 2,285 |
| ***V. vinifera*** | 30,298 | 14,705 | 26,796 | 3,430 | 3,502 |
| ***C. roseus*** | 30,062 | 13,939 | 25,993 | 5,298 | 4,069 |
| ***V. thouarsii*** | 27,143 | 13,078 | 25,672 | 4,880 | 1,471 |
| ***A. venetum*** | 18,559 | 14,672 | 18,547 | 45 | 12 |
| ***A. pictum*** | 19,898 | 14,921 | 19,588 | 174 | 310 |
| ***M. tenacissima*** | 21,899 | 14,044 | 21,152 | 649 | 747 |
| ***A. syriaca*** | 32,728 | 13,414 | 30,752 | 3,406 | 1,976 |
| ***C. gigatea*** | 18,197 | 13,648 | 17,601 | 156 | 596 |

**Supplementary Table S10. ApWRKY protein properties.**

| **Gene ID** | **Protein** | **Length (aa)** | **MW** | **pI** | **Instability**  **index** | **Aliphatic**  **index** | **GRAVY**  **score** |
| --- | --- | --- | --- | --- | --- | --- | --- |
| **g5491.t2** | **ApWRKY7** | 470 | 51,250.81 | 6.84 | 59.53 | 55.40 | −0.77 |
| **g5491.t1** | **ApWRKY6** | 526 | 57,617.36 | 7.74 | 61.13 | 48.23 | −0.96 |
| **g8620.t1** | **ApWRKY13** | 521 | 56,583.32 | 6.74 | 59.37 | 66.10 | −0.64 |
| **g16720.t1** | **ApWRKY40** | 737 | 80,662.65 | 5.74 | 55.33 | 56.26 | −0.74 |
| **g11877.t1** | **ApWRKY26** | 570 | 62,592.69 | 7.62 | 67.00 | 52.33 | −0.87 |
| **g13107.t1** | **ApWRKY28** | 571 | 62,909.82 | 6.90 | 65.25 | 50.23 | −0.91 |
| **g7707.t1** | **ApWRKY11** | 466 | 51,393.32 | 9.29 | 42.98 | 65.04 | −0.84 |
| **g6710.t1** | **ApWRKY10** | 508 | 56,029.43 | 8.40 | 61.38 | 57.78 | −0.99 |
| **g9133.t2** | **ApWRKY16** | 590 | 63,799.14 | 6.35 | 55.90 | 62.34 | −0.64 |
| **g9133.t1** | **ApWRKY15** | 547 | 59,375.21 | 6.30 | 58.45 | 60.79 | −0.67 |
| **g3031.t1** | **ApWRKY1** | 730 | 79,172.05 | 6.19 | 58.28 | 52.37 | −0.82 |
| **g11146.t1** | **ApWRKY25** | 267 | 30,642.40 | 8.05 | 53.71 | 49.66 | −1.08 |
| **g11146.t2** | **ApWRKY24** | 249 | 28,569.00 | 6.72 | 53.90 | 49.72 | −1.04 |
| **g9654.t1** | **ApWRKY18** | 588 | 65,405.65 | 7.61 | 52.08 | 61.97 | −0.98 |
| **g17002.t1** | **ApWRKY42** | 336 | 37,377.51 | 6.62 | 56.77 | 48.21 | −0.86 |
| **g5525.t1** | **ApWRKY8** | 330 | 35,454.80 | 5.54 | 62.59 | 42.58 | −0.81 |
| **g14009.t1** | **ApWRKY30** | 319 | 35,542.19 | 6.36 | 68.64 | 39.44 | −0.86 |
| **g11131.t1** | **ApWRKY23** | 318 | 35,470.72 | 8.64 | 63.68 | 53.62 | −0.72 |
| **g15119.t1** | **ApWRKY34** | 167 | 19,193.01 | 5.47 | 47.15 | 46.59 | −1.14 |
| **g4343.t1** | **ApWRKY5** | 182 | 21,085.59 | 8.19 | 58.65 | 51.37 | −0.99 |
| **g19414.t1** | **ApWRKY47** | 214 | 23,963.70 | 7.08 | 53.05 | 61.12 | −0.65 |
| **g4328.t1** | **ApWRKY4** | 220 | 25,322.46 | 9.21 | 60.15 | 58.05 | −0.86 |
| **g10609.t1** | **ApWRKY19** | 178 | 19,810.74 | 8.58 | 33.85 | 56.85 | −0.87 |
| **g15896.t1** | **ApWRKY35** | 183 | 20,974.85 | 9.49 | 48.68 | 72.46 | −0.77 |
| **g10935.t1** | **ApWRKY21** | 224 | 24,904.21 | 9.39 | 49.64 | 66.52 | −0.69 |
| **g5988.t1** | **ApWRKY9** | 178 | 20,276.88 | 9.51 | 52.61 | 60.67 | −0.80 |
| **g3190.t1** | **ApWRKY2** | 191 | 21,906.67 | 9.50 | 45.55 | 59.63 | −0.89 |
| **g17189.t1** | **ApWRKY43** | 380 | 41,047.70 | 5.46 | 59.83 | 49.37 | −0.84 |
| **g15898.t1** | **ApWRKY36** | 328 | 35,828.06 | 4.92 | 54.30 | 66.07 | −0.47 |
| **g10931.t1** | **ApWRKY20** | 353 | 38,935.01 | 5.79 | 65.78 | 50.06 | −0.90 |
| **g16937.t1** | **ApWRKY41** | 470 | 51,279.20 | 5.58 | 58.24 | 58.98 | −0.77 |
| **g14091.t1** | **ApWRKY31** | 272 | 29,228.28 | 5.50 | 48.63 | 61.32 | −0.56 |
| **g16146.t1** | **ApWRKY39** | 309 | 34,995.62 | 5.77 | 74.84 | 44.21 | −1.03 |
| **g14779.t1** | **ApWRKY49** | 330 | 35,848.63 | 9.76 | 43.93 | 63.24 | −0.60 |
| **g19202.t1** | **ApWRKY50** | 356 | 38,824.93 | 9.58 | 48.36 | 60.31 | −0.66 |
| **g11526.t1** | **ApWRKY48** | 344 | 38,394.57 | 9.46 | 54.54 | 71.13 | −0.67 |
| **g13336.t1** | **ApWRKY29** | 518 | 56,794.31 | 6.26 | 57.08 | 66.12 | −0.77 |
| **g8683.t1** | **ApWRKY14** | 446 | 48,451.19 | 8.75 | 54.16 | 62.60 | −0.62 |
| **g14474.t1** | **ApWRKY32** | 629 | 67,932.28 | 6.34 | 39.54 | 63.08 | −0.64 |
| **g18399.t1** | **ApWRKY46** | 576 | 62,364.47 | 6.74 | 42.15 | 62.05 | −0.63 |
| **g9204.t1** | **ApWRKY17** | 535 | 58,787.73 | 6.61 | 42.27 | 64.45 | −0.68 |
| **g3628.t1** | **ApWRKY3** | 324 | 35,879.24 | 8.35 | 46.01 | 63.24 | −0.77 |
| **g14842.t1** | **ApWRKY33** | 314 | 34,659.09 | 6.71 | 50.13 | 72.71 | −0.59 |
| **g7814.t1** | **ApWRKY12** | 295 | 33,611.57 | 5.60 | 59.87 | 68.75 | −0.81 |
| **g13050.t1** | **ApWRKY27** | 310 | 34,804.64 | 6.09 | 50.75 | 70.77 | −0.65 |
| **g17942.t1** | **ApWRKY45** | 330 | 37,210.07 | 8.02 | 46.00 | 69.42 | −0.64 |
| **g10990.t1** | **ApWRKY22** | 360 | 40,281.06 | 4.90 | 45.24 | 70.69 | −0.59 |
| **g15962.t2** | **ApWRKY38** | 322 | 36,196.01 | 6.15 | 56.85 | 62.73 | −0.77 |
| **g15962.t1** | **ApWRKY37** | 360 | 40,392.47 | 5.63 | 55.74 | 61.25 | −0.74 |
| **g17755.t1** | **ApWRKY44** | 360 | 39,882.23 | 6.36 | 57.70 | 69.92 | −0.65 |

**Figures**


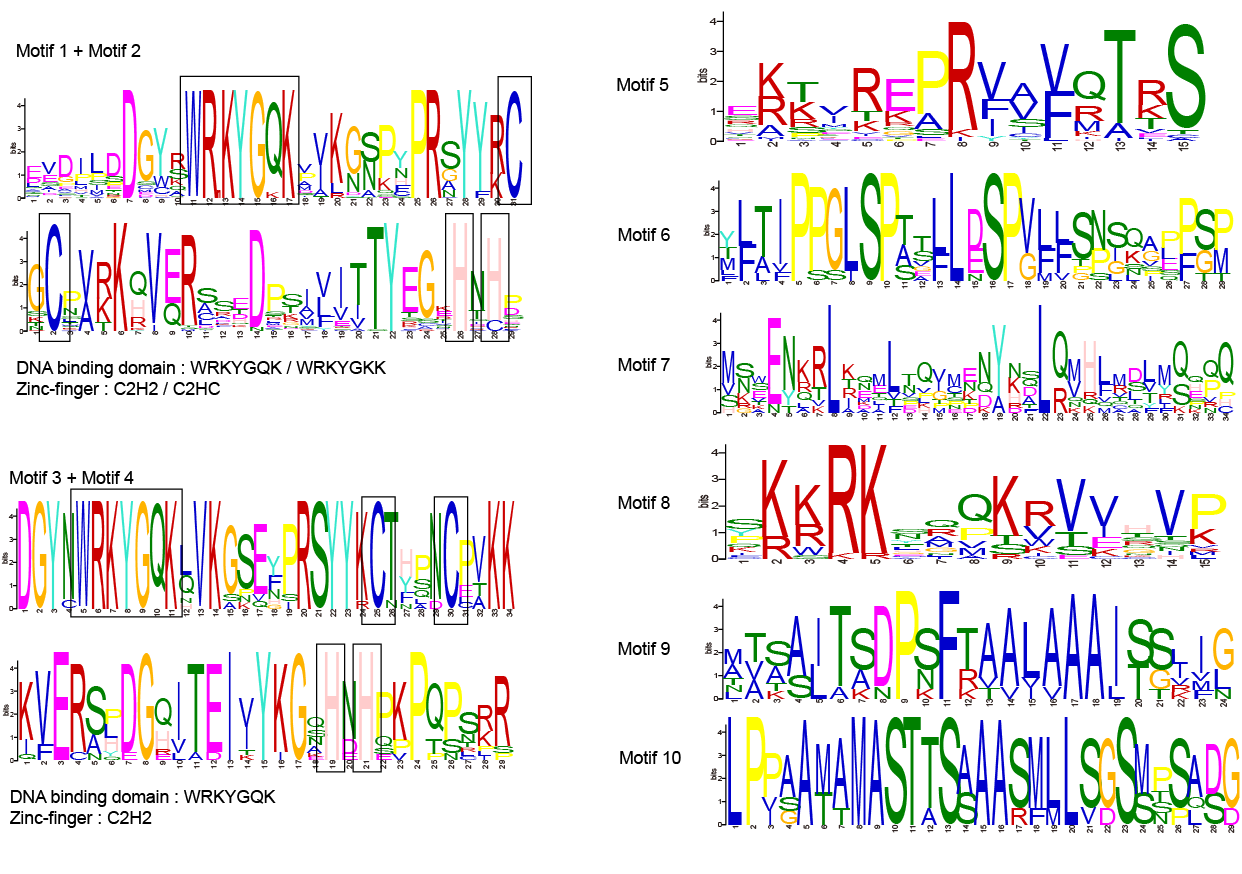


**Supplementary Figure S1.** Conserved motifs of the ApWRKY proteins. The two major WRKY domains consisted of motifs 1 and 2 and motifs 3 and 4, respectively.


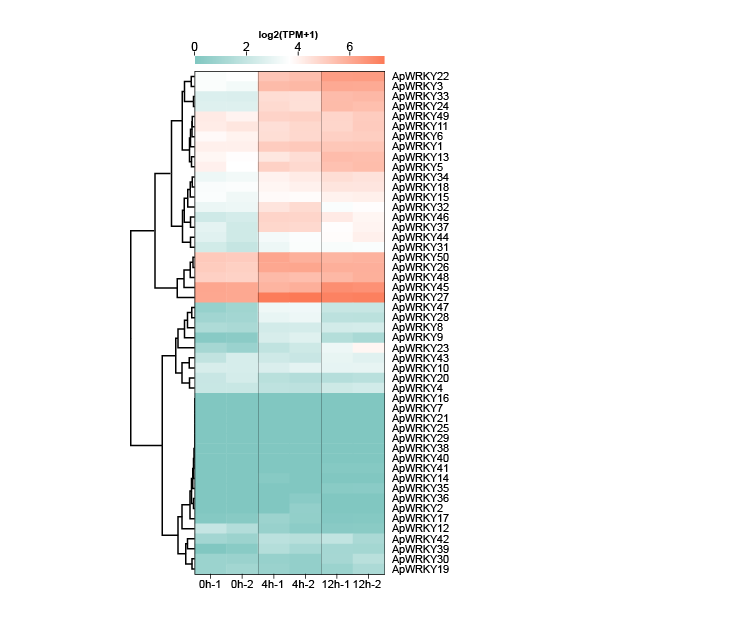


**Supplementary Figure S2.** Expression profiles of the *ApWRKY* genes in leaves subjected to 0, 4, or 12 h of drought stress. The colored scale from green to red indicates increasing expression level.
